# Prevalence and factors associated with treatment delay among colorectal cancer patients at Mulago National Referral Hospital and the Uganda Cancer Institute: A cross-sectional study

**DOI:** 10.1371/journal.pone.0353668

**Published:** 2026-07-17

**Authors:** Brian Kasagga, Paul Ssempebwa, Godfrey Kikuba, Flavius E. Egbe, Peace Caroline Nsodi, Joanne Kayaga, Emmanuel Alex Elobu, Paul Okeny

**Affiliations:** 1 Department of Surgery, School of Medicine, College of Health Sciences, Makerere University, Kampala, Uganda; 2 Department of Surgery, Mulago National Referral Hospital, Kampala, Uganda; 3 Department of Gastrointestinal Oncology, Uganda Cancer Institute, Kampala, Uganda; Makerere University College of Health Sciences, UGANDA

## Abstract

**Background:**

Colorectal cancer (CRC) is an important cause of morbidity and mortality in Uganda. Timely treatment initiation is critical for outcomes, yet delays are common. This study assessed treatment delays and associated factors among CRC patients at Mulago National Referral Hospital (MNRH) and the Uganda Cancer Institute (UCI).

**Objective:**

To determine the diagnosis to treatment interval (DTI), prevalence of treatment delay, and the associated patient and clinicopathologic factors among CRC patients.

**Methods:**

A hospital-based cross-sectional study was conducted among 67 patients with histologically confirmed CRC between December 2024 and May 2025. Treatment delay was defined as >31 days between histological diagnosis and first oncologic treatment. Data were collected through interviews and record review. Descriptive statistics summarized demographics and clinical characteristics. Bivariate Poisson regression with robust variance estimation identified factors associated with delay; variables with p < 0.20 entered a multivariable model. Prevalence ratios (PRs) with 95% levels of confidence were reported. IRB approval was obtained (Ref: Mak-SOMREC-2024-1048).

**Results:**

The mean age was 50.5 years (SD: 15.1); 55.2% were female, and 71.6% (n = 48) had advanced-stage disease (Stage III/IV). The median DTI was 53 days (IQR: 25–95), with 70.1% (n = 47) experiencing delays. Median DTI by treatment: chemotherapy 53 days, radiotherapy 79 days, surgery 14 days. Socioeconomic vulnerability was the only factor independently associated with treatment delay. Patients with high socioeconomic vulnerability scores (≥4) had a 34% higher prevalence of delay (PR = 1.34, 95% CI: 1.01–1.78, p = 0.042).

**Conclusion:**

Most CRC patients experienced treatment delays which were widespread and occurred across all categories; regardless of distance to the treatment facility, clinical status, or disease severity. Socioeconomic disadvantage was the only independent predictor, underscoring the role of structural and financial barriers in timely care. Targeted, context-specific interventions are urgently needed to reduce delays and improve outcomes.

**Trial registration:**

Not applicable.

## Background

Colorectal cancer (CRC) represents a major global public health burden, ranking as the third most commonly diagnosed malignancy and the second leading cause of cancer-related mortality worldwide [1]. In 2020, an estimated 1.9 million new CRC cases and 930,000 deaths were reported, with projections suggesting a 63% increase in incidence (3.2 million cases annually) and a 73% rise in mortality (1.6 million deaths annually) by 2040 [2].

Although the incidence of CRC has historically been lower in Africa compared to high-income regions, there is growing concern over a rising trend, particularly in Sub-Saharan Africa (SSA). Lifestyle changes, increasing urbanization, and dietary shifts towards processed foods have contributed to a growing incidence [3,4]. In 2019, SSA recorded approximately 58,000 new CRC cases, 49,000 deaths, and over 1.3 million disability-adjusted life years (DALYs), underscoring both the growing burden and the limitations in health system capacity to manage it [5]. The opposite demographic trend has been observed in high income countries due to robust screening programs and therapeutic advances [3,4].

In Uganda, CRC accounts for an increasing share of the national cancer burden. According to the Kampala Cancer Registry, incidence rates have nearly tripled between 2008 and 2021 [6,7]. CRC now ranks as the eighth most commonly diagnosed cancer in Uganda, with an estimated 1,394 new cases and 1,013 deaths annually. The 5-year prevalence stands at approximately 3,000 cases, corresponding to 6.2 per 100,000 population [8,9]. However, data on survival outcomes remain limited due to challenges in cancer surveillance, late diagnosis, and underreporting.[7] Health system constraints such as limited endoscopy services, pathology capacity, surgical expertise, and oncology infrastructure further exacerbate delays in diagnosis and treatment [10]. These systemic barriers, common across many low- and middle-income countries (LMICs), result in significantly prolonged intervals from symptom onset to definitive treatment—often 1.5 to 4 times longer than in high-income countries (HICs) [11].

Evidence consistently shows that treatment delays are associated with poorer clinical outcomes in CRC patients [12–15]. This is because delays contribute to tumor progression, increased risk of micro-metastatic disease, and reduced treatment efficacy [16]. However, treatment delay is only one of several interrelated factors that influence survival. Tumor biology, including genetic and molecular characteristics such as microsatellite instability and KRAS/BRAF mutations;- also plays a major role in prognosis and therapeutic response [17]. Additionally, patient-related factors like comorbidities, performance status (e.g., ECOG score), and socioeconomic conditions—including access to care, education, and financial capacity—can significantly affect treatment outcomes [18,19]. Psychological distress and prolonged uncertainty may further compromise a patient’s ability to manage their illness [20].

Treatment delays necessitate more extensive interventions due to disease progression, compromises quality of life, and increases direct healthcare costs [21]. Although multiple factors influence outcomes, these delays are potentially modifiable contributors—especially in low-resource settings like Uganda. Despite this recognition, there is limited context-specific evidence in Uganda quantifying diagnosis-to-treatment intervals and identifying factors associated with delayed treatment initiation. Existing studies have largely focused on describing care pathways rather than systematically examining patient-level, clinicopathologic, and socioeconomic determinants of treatment delay for CRC.[22].

Understanding these delays and their context-specific drivers is essential for improving timely access to care and, ultimately, enhancing patient survival. This study therefore aims to determine the interval from diagnosis to treatment initiation and to identify factors associated with treatment delays among CRC patients in MNRH and UCI.

## Methods

### Study design and setting

We conducted a retrospective hospital-based cross-sectional study at Mulago National Referral Hospital (MNRH) and the Uganda Cancer Institute (UCI) in Kampala, Uganda, between December 2024 and May 2025. During this period, patients who had already initiated first oncologic treatment were identified, and relevant diagnostic and treatment dates were retrospectively reviewed. These two public tertiary facilities jointly manage CRC patients through an integrated referral and multidisciplinary care pathway. The diagnosis-to-treatment interval was reconstructed for each participant, and the study focused on estimating this interval and the prevalence of treatment delay.

MNRH is Uganda’s principal public tertiary referral hospital, with a bed capacity of approximately 1,500 and over 650,000 patient visits annually, including more than 14,000 surgical admissions. It serves as the main centre for diagnostic evaluation, endoscopy, histopathological confirmation, and surgical management of CRC. The Uganda Cancer Institute is the national public cancer referral centre, receiving an estimated 4,000 new cancer patients annually and providing specialised oncological services including chemotherapy and radiotherapy. Most public-sector CRC patients receive care across both institutions, which serve a national catchment area and conduct joint multidisciplinary tumour boards for coordinated treatment planning.

### Study population and sampling

The study population comprised adult patients (≥18 years) with histologically confirmed CRC receiving care at Mulago National Referral Hospital (MNRH) and/or the Uganda Cancer Institute (UCI). Eligible participants were required to have initiated first oncologic treatment (surgery, chemotherapy, or radiotherapy). Patients who had not commenced treatment, had missing or indeterminate diagnosis or treatment dates, or declined consent were excluded.

Sample size determination was guided by the study objectives. To estimate the prevalence of treatment delay, the Kish–Leslie single population proportion formula was applied, assuming a 95% confidence level, an expected prevalence of 43% based on prior literature, and a 5% margin of error. Hospital records indicated that approximately 158 CRC patients are diagnosed annually across MNRH and UCI, yielding an accessible population of about 79 patients over the six-month study period. A finite population correction was therefore applied, resulting in a minimum required sample size of 67 participants.

For analysis of factors associated with treatment delay, sample size was calculated using the Fleiss formula for comparison of proportions, based on the primary exposure variables of interest. The final sample size was taken as the larger of the estimates derived for the prevalence and factors objectives (67). Participants were enrolled using consecutive sampling during routine clinical care encounters, including surgical wards and outpatient clinics at MNRH and oncology clinics, chemotherapy units, and radiotherapy services at UCI. Given the integrated referral pathway and frequent patient movement between the two institutions, strict proportionate sampling by site was not operationally feasible, and enrolment reflected real-world patient flow across the CRC care continuum.

### Data collection

Data were collected using structured patient interviews and medical record review. Patient interviews were used to capture information not routinely documented in clinical records, while medical record review provided clinical and treatment-related data required for the study objectives. These sources were used in a complementary manner, with interviews supplementing records where documentation was incomplete.

Face-to-face interviews were conducted by trained research assistants using a structured questionnaire informed by prior literature on delays in cancer care in low-resource settings. The questionnaire captured sociodemographic characteristics, health-seeking pathways prior to diagnosis, referral experiences, and use of alternative or traditional medicine.

Medical record review was conducted using a standardized data abstraction form. Inpatient and outpatient records at Mulago National Referral Hospital and oncology clinic records at the Uganda Cancer Institute were reviewed to extract dates of histological diagnosis and treatment initiation, clinical stage at diagnosis, and type of initial treatment received. Interview data were cross-checked against available records where applicable.

### Data management and statistical analysis

Data were collected through patient interviews and medical record reviews using a pre-tested, structured questionnaire. The primary outcome was treatment delay, defined as an interval of >31 days from histopathological confirmation to the first oncologic treatment [23].

Data were analyzed using SPSS version 20. Descriptive statistics were used to summarize participant characteristics. The prevalence of delay was reported as a proportion with a 95% level of confidence.

#### Bivariate and Multivariable Regression.

Bivariate analysis was performed using Poisson regression with robust variance to estimate crude Prevalence Ratios (PR) for factors associated with treatment delay. Education categories were collapsed prior to regression analyses to improve statistical stability due to small cell sizes in higher education categories. Variables with a p-value < 0.2 were included in a multivariable Poisson regression model to generate adjusted Prevalence Ratios (aPR) with 95% CIs. A p-value < 0.05 was considered statistically significant.

#### Development of Composite Scores.

Recognizing the multidimensional nature of barriers to care, we developed three composite scores post-hoc to better capture complex constructs:

Socioeconomic Status (SES) Score (Range: 0–7): A cumulative score of disadvantage, incorporating employment status (unemployed = 2, previously employed = 1, employed = 0), education level (none = 2, primary = 1, secondary+=0), marital status (widowed/divorced = 2, single = 1, married = 0), and health behaviors (smoking = 1, alternative medicine use = 1). A higher score indicates greater socioeconomic vulnerability.

Disease Severity Score (Range: 2–8): Sum of tumor stage (I = 1, II = 2, III = 3, IV = 4) and tumor grade (1 = 1, 2 = 2, 3 = 3, 4 = 4).

Clinical Burden Score (Range: 0–6): Sum of the presence of comorbidities (yes = 1), ECOG performance status (0–4), and low BMI < 18.5 kg/m² (yes = 1).

For analysis, the total scores for SES and Clinical Burden were dichotomized into ‘Low’ and ‘High’ burden based on the distribution of the data. Composite scores were constructed as exploratory summaries of related barriers to reduce dimensionality and facilitate interpretation and were not intended to replace analyses of individual factors associated with treatment delay. This is further illustrated in Table 1 of the Supplementary file. The association between these composite scores and treatment delay was then analyzed using the same regression framework described above.

**Table 1:** Baseline characteristics of participants.

Characteristic	Category	n (%) or Median (IQR)
Age (years)	–	56 (41–62)
BMI (kg/m²)	–	21.6 (19.4–24.3)
Distance to facility (km)	–	137.0 (52.0–327.7)
Sex	Male	30 (44.8%)
	Female	37 (55.2%)
Education level	No formal education	5 (7.5%)
	Primary	30 (44.8%)
	Secondary	18 (26.9%)
	College/University	12 (17.9%)
	Postgraduate	2 (3.0%)
Employment status	Unemployed	16 (23.9%)
	Employed	11 (16.4%)
	Private work	34 (50.7%)
	Employed but not currently working	6 (9.0%)
Marital status	Married	47 (70.1%)
	Single	6 (9.0%)
	Divorced	4 (6.0%)
	Widowed	10 (14.9%)
Religion	Protestant	27 (40.3%)
	Catholic	19 (28.4%)
	Muslim	7 (10.4%)
	Baptist	13 (19.4%)
	Born again	1 (1.5%)
Smoking status	No	59 (88.1%)
	Yes	8 (11.9%)
Tumor location	Colon	21 (31.3%)
	Rectal	42 (62.7%)
	Rectosigmoid	4 (6.0%)
Tumor grade	Grade 1	26 (38.8%)
	Grade 2	26 (38.8%)
	Grade 3	13 (19.4%)
	Grade 4	2 (3.0%)
Clinical stage	Stage I	4 (6.0%)
	Stage II	12 (17.9%)
	Stage III	32 (47.8%)
	Stage IV	16 (23.9%)
	Missing data**	3 (4.5%)
Comorbidities	No	50 (74.6%)
	Yes	17 (25.4%)
ECOG status	0	16 (23.9%)
	1	41 (61.2%)
	2	10 (14.9%)
First oncologic treatment	Surgery	5 (7.5%)
	Chemotherapy	55 (82.1%)
	Radiotherapy	7 (10.4%)
Pretreatment CEA	Normal	29 (43.3%)
	Elevated	17 (25.4%)
	High	21 (31.3%)

**Clinical stage data were missing for three patients due to incomplete documentation in the medical records at the time of data abstraction.

### Ethical considerations

The study was approved by the Makerere University School of Medicine Research and Ethics Committee (Ref: Mak-SOMREC-2024-1048) and administrative clearance was obtained from both hospitals. Written consent was obtained from all participants after detailed explanation of the study objectives and procedures.

## Results

A total of 67 patients with histologically confirmed CRC were included. Overall, the cohort was characterised by a predominance of advanced-stage disease, rectal tumour location, and good functional status at presentation. Most patients received systemic chemotherapy as first-line oncologic treatment. The median distance from a patient’s residence to the treatment facility was 137.0 km (IQR: 52.0–327.7). Detailed demographic and clinical characteristics are presented in Table 1.

### Treatment interval and prevalence of delay

**Fig 1:**
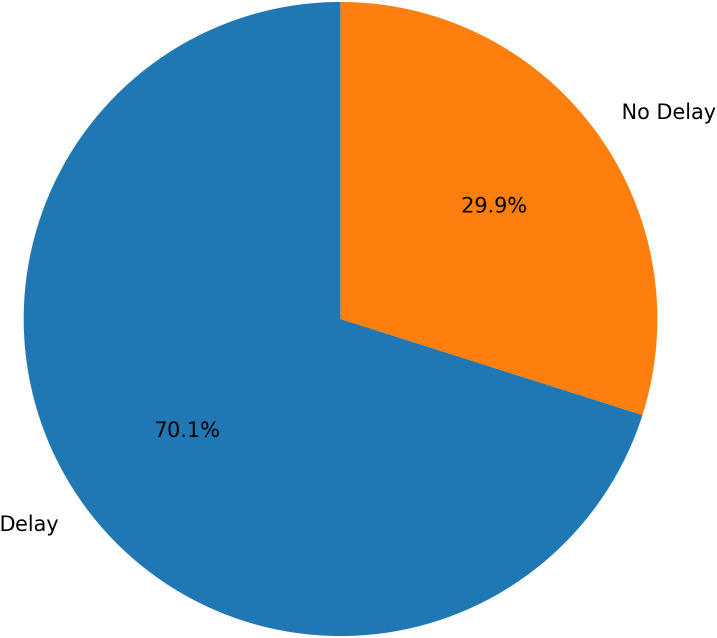
Prevalence of treatment delay among colorectal cancer patients.

The median time from histological diagnosis to initiation of the first oncologic treatment was 53 days (IQR: 25–95 days), with a wide range from 3 to 1,133 days. The treatment interval for surgery was (median: 14 days; IQR: 6.5–36.0), chemotherapy (median: 53 days; IQR: 27.0–96.0), and radiotherapy (median: 79 days; IQR: 46.0–110.0). A detailed breakdown of the treatment interval across all patient subgroups is provided in Supplementary Table 2**.** Applying the pre-defined threshold of >31 days, the prevalence of treatment delay among CRC patients at MNRH and UCI was 70.1% (47/67, 95% CI: 58.5% − 81.7%). Only 29.9% (20/67) of patients initiated treatment within 31 days (Fig 1).

### Factors associated with treatment delay

#### Bivariate analysis of individual factors.

Bivariate analysis using Poisson regression with robust variance was performed to assess the association between various patient, clinical, and pathological factors and treatment delay. The complete results are presented in Table 2.

**Table 2:** Bivariate analysis of factors associated with treatment delay (N = 67).

Variable	Category	PR	95% CI	p-value
**Sex**	Male (Ref: Female)	0.91	0.66–1.26	0.580
**Age**	<50 yrs (Ref: ≥ 50 yrs)	0.84	0.59–1.20	0.337
**Distance to facility**	≥200 km (Ref: < 200 km)	0.88	0.65–1.19	0.403
**Comorbidities**	Yes (Ref: No)	0.80	0.60–1.08	0.144
Education Level*	Primary or less	0.98	0.71–1.36	0.913
	Secondary	0.64	0.37–1.09	0.099
	Tertiary level (University/College/ Postgraduate); (**Ref: Tertiary)**	**1.00**	**–**	**–**
**Marital Status**	Married	0.85	0.59–1.23	0.388
	Single	0.83	0.44–1.59	0.580
	Divorced	0.94	0.49–1.79	0.845
	**(Ref: Widowed)**	**1.00**	**–**	**–**
**Smoking History**	Non-smoker (Ref: Smoker)	0.88	0.50–1.54	0.649
**Alternative Therapy**	No (Ref: Yes)	0.94	0.66–1.35	0.751
**Tumor Location**	Colon	1.33	0.48–3.72	0.582
	Rectal	1.48	0.55–4.00	0.444
	**(Ref: Rectosigmoid)**	**1.00**	**–**	**–**
**Tumor Grade**	Grade 1	1.31	0.32–5.38	0.710
	Grade 2	1.54	0.38–6.25	0.547
	Grade 3	1.38	0.33–5.80	0.656
	**(Ref: Grade 4)**	**1.00**	**–**	**–**
**Clinical Stage**	Stage I	1.13	0.59–2.16	0.724
	Stage II	0.88	0.49–1.56	0.651
	Stage III	1.14	0.78–1.66	0.509
	**(Ref: Stage IV)**	**1.00**	**–**	**–**
**ECOG Status**	0	1.07	0.65–1.76	0.785
	1	0.98	0.62–1.54	0.916
	**(Ref: 2)**	**1.00**	**–**	**–**

PR: Prevalence Ratio; CI: Confidence Interval; Ref: Reference category * Education was reclassified into three categories (primary or less, secondary, and tertiary [college/university or postgraduate]) to improve statistical stability due to small cell sizes in higher education categories.

After collapsing education categories to improve statistical stability, education level was not significantly associated with treatment delay in bivariate analysis (Table 2). No other demographic, clinical, or pathological factors showed a statistically significant association with treatment delay.

#### Analysis using composite scores.

To address the limitations of interpreting education level in isolation and to summarise related barriers to care, we constructed three composite scores representing socioeconomic vulnerability, disease severity, and clinical burden (scoring details provided in Supplementary Table 2). These composite scores were used as exploratory measures to capture the multidimensional nature of barriers to treatment initiation.

In bivariate analyses, higher socioeconomic vulnerability was associated with an increased prevalence of treatment delay, whereas disease severity and clinical burden composite scores were not significantly associated with delay (Table 3).

**Table 3:** Bivariate analysis of composite scores associated with treatment delay.

Variable	Category	PR	95% CI	p-value
**SES Composite Score**	High (Ref: Low)	1.38	1.06–1.79	**0.020**
**Disease Severity Score**	High (Ref: Low)	1.13	0.83–1.53	0.454
**Clinical Burden Score**	High (Ref: Low)	0.84	0.62–1.13	0.247

#### Final adjusted model.

In the final multivariable model, the socioeconomic vulnerability composite score remained independently associated with treatment delay after adjustment for age, sex, and distance to the treatment facility. Patients with high socioeconomic vulnerability had a 34% higher prevalence of delay compared to those with lower vulnerability (aPR = 1.34, 95% CI: 1.01–1.78, p = 0.042). No significant associations were observed for age, sex, or distance in the final model (Table 4).

**Table 4:** Final multivariable model of factors associated with treatment delay.

Variable	Category	aPR	95% CI	p-value
**SES Composite Score**	High (Ref: Low)	1.34	1.01–1.78	**0.042**
**Age Group**	<50 yrs (Ref: ≥ 50)	0.89	0.64–1.26	0.518
**Sex**	Male (Ref: Female)	0.98	0.71–1.35	0.888
**Distance to facility**	(per km)	1.00	0.999–1.001	0.970

aPR: Adjusted Prevalence Ratio.

## Discussion

This study sought to determine the prevalence of treatment delays and associated factors among CRC patients at Mulago National Referral Hospital and the Uganda Cancer Institute. Treatment delays were common in this setting, with diagnosis-to-treatment intervals frequently exceeding most recommended benchmarks and society guidelines. While most individual demographic, clinical, and pathological factors were not independently associated with delay, socioeconomic vulnerability emerged as an important determinant, with patients experiencing higher socioeconomic vulnerability showing a greater likelihood of delayed treatment initiation. When compared to previous studies and against international benchmarks, our findings reveal substantial disparities in timely oncologic CRC cancer care. Compared to recommended treatment intervals of 2–4 weeks for most common cancers Uganda’s median delay of 53 days represents a significant deviation from global comparisons [11,24]. This delay is considerably longer than intervals reported in other settings; for instance, Bouter et al. in South Africa reported a median diagnosis-to-treatment interval of 29 days among CRC patients [25], while studies from Poland and Italy report averages of 38 days and median of 28 days respectively [26,27]. These discrepancies likely reflect variations in healthcare infrastructure and access to timely care between different resource settings [21].

Within the Ugandan context, our findings both align and contrast with previous research. Kibudde et al., examining treatment intervals across various cancers, reported a median waiting time of 33 days (range: 16–416) [28], which was numerically lower, but within a comparable range. The overall median therefore reflected shorter treatment times associated with other malignancies, such as cervical, head and neck, sarcoma, and esophageal cancers, which were more prevalent in that cohort. Furthermore, CRC was underrepresented in their study, with only one patient included [28]. In contrast, our study focused exclusively on CRC, which may follow different treatment pathways. However, our findings regarding modality-specific delays show concerning consistency with local patterns; similar to Kibudde et al., we found substantially longer turnaround times for radiotherapy (median 79 days) and chemotherapy (median 53 days). This disparity reflects the additional complexities of radiotherapy planning and systemic challenges including limited equipment availability and fragmented care coordination [29,30].

The prevalence of treatment delay in our study (70.1%) exceeds rates reported in other African settings, including Ethiopia (43%) and Botswana (50.4%), though methodological differences in defining delay thresholds must be acknowledged [23,31]. This high prevalence underscores the profound systemic challenges within Uganda’s oncology infrastructure, characterized by limited radiotherapy capacity, diagnostic bottlenecks, and centralized care concentrated in Kampala [10,32]. The biological implications of these delays are particularly concerning given tumor doubling time in CRC ranges from 92 to over 1032 days [33]; our mean interval of 100 days represents a period sufficient for meaningful tumor progression, potentially compromising curative outcomes, especially for over 71.6% of patients who already present with advanced-stage disease [34].

Our analysis of associated factors revealed that socioeconomic vulnerability, captured through a multidimensional composite score, was the only independent predictor of treatment delay. Although education level alone was not independently associated with treatment delay after reclassification, this finding underscores the limitations of interpreting single socioeconomic indicators in isolation. When education was considered as part of a broader socioeconomic vulnerability composite, which incorporated employment status, marital status, and health-related behaviors, cumulative socioeconomic disadvantage emerged as a significant predictor of treatment delay. This suggests that in resource-constrained settings such as Uganda, structural and intersecting socioeconomic barriers, rather than individual characteristics, play a more decisive role in determining timely access to CRC care [35,36].

In this study, use of alternative or traditional medicine was conceptualised as a marker of socioeconomic vulnerability rather than a purely cultural or clinical factor. In the Ugandan context, reliance on alternative medicine is often driven by structural constraints such as financial barriers, geographic inaccessibility of biomedical services, and delayed access to formal health care, rather than cultural preference alone [37,38]. As such, inclusion of alternative medicine use in the SES composite reflects constrained access to timely, affordable biomedical care, which is directly relevant to treatment delay.

The consistent relationship between socioeconomic disadvantage and treatment delay aligns with findings from other settings across sub-Saharan Africa. Buckle et al. in Kenya reported longer waiting times among patients from rural and lower-income backgrounds [39] while Wassie et al. in Ethiopia linked delays to financial hardship and lack of awareness [23]. Even in high-income settings, composite SES indices have predicted disparities in cancer care timelines [18,40], highlighting the universal influence of structural inequality on health outcomes. Our study contributes to this literature by demonstrating the particular utility of multidimensional SES measures in low-resource settings where traditional income-based metrics may fail to capture the complexity of socioeconomic vulnerability.

Several limitations should be considered when interpreting these findings. Although the study was conducted at the two principal public referral centres for CRC care in Uganda, the sample size was modest which limited the power for subgroup analyses. Although education categories were reclassified to improve stability, residual misclassification is possible, and findings related to individual socioeconomic indicators should be interpreted cautiously. Consecutive sampling, a non-probability sampling technique, was employed and may have introduced selection bias, potentially limiting the generalisability of the findings beyond similar public-sector referral settings. In addition, the socioeconomic vulnerability composite score was developed post hoc as an exploratory measure and requires validation in larger, independent cohorts. Finally, some potentially relevant influences on treatment delay, including detailed health system processes, cultural beliefs, and individual health-seeking behaviours, were not fully captured and may have contributed to delays observed in this study.

In conclusion, this study demonstrates that treatment delays are pervasive among CRC patients managed at Uganda’s main public referral hospitals, with socioeconomic vulnerability emerging as the primary determinant of delayed care. Diagnosis-to-treatment intervals substantially exceed international benchmarks and likely contribute to the poor outcomes observed among Ugandan CRC patients. These findings highlight the need for multi-level interventions that address structural barriers and system inefficiencies, including improved care coordination, financial protection mechanisms, and routine monitoring of treatment timelines. Prioritising socioeconomically vulnerable patients and establishing time-to-treatment as a quality indicator may be critical steps in strengthening oncology care delivery in Uganda.

## Supporting information

S1 Table Definition and stratification of the socioeconomic vulnerability, disease severity, and clinical burden composite scores. (DOCX)

S2 Table Treatment intervals across patient demographic and clinicopathologic subgroups. (DOCX)

## Abbreviations

CRC: Colorectal Cancer
DTI: Diagnosis to Treatment Interval
MDT: multidisciplinary team
MNRH: Mulago National Referral Hospital
PI: Principal Investigator
SES: Socioeconomic Status
SOMREC: School of Medicine Research and Ethics Committee
TI: treatment interval
TNM: Tumor, Nodal, Metastasis
UCI: Uganda Cancer Institute.
