# Supplementary material for: Prevalence and factors associated with treatment delay among colorectal cancer patients at Mulago National Referral Hospital and the Uganda Cancer Institute: A cross-sectional study
Source: PLoS One. 2026 Jul 17;21(7):e0353668. doi: 10.1371/journal.pone.0353668 (PMC13378963; doi:10.1371/journal.pone.0353668)
Supplement: S1 Table — (DOCX) [file pone.0353668.s001.docx]

**Table 1: Composite score definition and stratification**

| **Composite Score** | **Components** | **Points Assigned** | **Total Range** | **Stratification** |
| --- | --- | --- | --- | --- |
| **Socioeconomic (SES)** | Employment: | Unemployed=2, Previously employed=1, Private employment or Employed=0 | 0–7 | Low (1–3), High (4–7) |
|  | Education: | No formal=2, Primary=1, Secondary+=0 |  |  |
|  | Marital Status: | Widowed/Divorced=2, Single=1, Married=0 |  |  |
|  | Health Behaviors: | Smoking=1, Alternative medicine=1 |  |  |
| **Disease Severity** | Tumor Stage: | I=1, II=2, III=3, IV=4 | 2–8 | Low (≤4), High (≥5) |
|  | Tumor Grade: | 1=1, 2=2, 3=3, 4=4 |  |  |
| **Clinical Burden** | Comorbidities: | Present=1, Absent=0 | 0–6 | Low (≤2), High (≥3) |
|  | ECOG Status: | 0=0, 1=1, 2=2, 3=3, 4=4 |  |  |
|  | Low BMI (<18.5 kg/m²): | Yes=1, No=0 |  |  |
