# Supplementary material for: Prevalence and factors associated with treatment delay among colorectal cancer patients at Mulago National Referral Hospital and the Uganda Cancer Institute: A cross-sectional study
Source: PLoS One. 2026 Jul 17;21(7):e0353668. doi: 10.1371/journal.pone.0353668 (PMC13378963; doi:10.1371/journal.pone.0353668)
Supplement: S2 Table — (DOCX) [file pone.0353668.s002.docx]

**Table 2: Treatment interval between different subgroups**

| **Variable** | **Category** | **N** | **Mean ± SD** | **Median [IQR]** | **Range** |
| --- | --- | --- | --- | --- | --- |
| **Sex** | Male | 30 | 67.1 ± 67.3 | 45.5 [21.8–83.3] | 7–294 |
|  | Female | 37 | 126.9 ± 226.8 | 63.0 [26.0–101.0] | 3–1133 |
| **Highest Education** | No formal education | 5 | 166.2 ± 162.5 | 95.0 [27.5–340.5] | 13–362 |
|  | Primary | 30 | 93.8 ± 154.4 | 54.5 [32.3–98.0] | 10–861 |
|  | Secondary | 18 | 61.9 ± 70.9 | 34.5 [18.5–79.0] | 3–294 |
|  | College/University | 12 | 151.3 ± 313.0 | 66.0 [20.8–93.8] | 7–1133 |
|  | Postgraduate | 2 | 66.0 ± 24.0 | 66.0 [49.0–83.0] | 49–83 |
| **Marital Status** | Married | 47 | 69.3 ± 70.4 | 49.0 [23.0–83.0] | 3–362 |
|  | Single | 6 | 68.3 ± 64.4 | 53.0 [14.8–117.8] | 8–183 |
|  | Divorced | 4 | 312.0 ± 547.5 | 45.0 [27.5–863.5] | 25–1133 |
|  | Widowed | 10 | 179.3 ± 258.7 | 79.0 [32.3–250.0] | 13–861 |
| **Religion** | Protestant | 27 | 70.0 ± 50.5 | 64.0 [25.0–95.0] | 3–186 |
|  | Catholic | 19 | 69.4 ± 88.1 | 42.0 [23.0–61.0] | 7–319 |
|  | Muslim | 7 | 174.9 ± 305.0 | 59.0 [27.0–131.0] | 15–861 |
|  | Baptist | 13 | 174.2 ± 305.2 | 63.0 [25.5–189.0] | 13–1133 |
|  | Born again | 1 | *Single value* | *Single value* | *Single value* |
| **Smoking** | No | 59 | 106.7 ± 186.3 | 53.0 [23.0–96.0] | 3–1133 |
|  | Yes | 8 | 51.8 ± 34.9 | 34.5 [27.3–83.0] | 14–110 |
| **Alternative Therapy** | No | 52 | 109.4 ± 194.0 | 57.0 [23.5–97.5] | 3–1133 |
|  | Yes | 15 | 68.1 ± 86.1 | 45.0 [28.0–83.0] | 7–362 |
| **Tumor Location** | Colon | 21 | 114.7 ± 240.0 | 63.0 [24.0–91.5] | 3–1133 |
|  | Rectal | 42 | 97.9 ± 145.5 | 53.0 [26.0–99.5] | 10–861 |
|  | Rectosigmoid | 4 | 46.8 ± 48.2 | 31.0 [12.8–96.5] | 8–117 |
| **Grade** | 1 | 26 | 88.5 ± 93.1 | 61.0 [22.0–120.5] | 14–362 |
|  | 2 | 26 | 72.0 ± 63.1 | 57.0 [31.0–83.3] | 8–294 |
|  | 3 | 13 | 107.3 ± 228.5 | 45.0 [20.0–75.0] | 3–861 |
|  | 4 | 2 | 570.0 ± 796.2 | 570.0 [7.0–1133.0] | 7–1133 |
| **Clinical Stage** | 1 | 4 | 119.5 ± 75.5 | 136.0 [41.3–181.3] | 23–183 |
|  | 2 | 12 | 86.9 ± 106.0 | 43.0 [20.3–83.0] | 10–319 |
|  | 3 | 33 | 72.1 ± 72.0 | 53.0 [30.0–96.5] | 3–362 |
|  | 4 | 18 | 155.9 ± 311.6 | 55.5 [16.3–85.0] | 7–1133 |
| **Comorbidities** | No | 50 | 106.0 ± 198.7 | 50.0 [18.8–85.0] | 3–1133 |
|  | Yes | 17 | 82.7 ± 80.1 | 53.0 [39.5–107.0] | 13–362 |
| **ECOG** | 0 | 16 | 85.0 ± 76.9 | 62.0 [29.0–122.8] | 13–294 |
|  | 1 | 41 | 113.5 ± 217.6 | 49.0 [23.0–86.0] | 3–1133 |
|  | 2 | 10 | 69.4 ± 66.5 | 50.0 [12.3–101.3] | 8–227 |
| **First Oncologic Treatment** | Surgery | 5 | 19.8 ± 17.9 | 14.0 [6.5–36.0] | 3–49 |
|  | Chemotherapy | 55 | 110.1 ± 192.0 | 53.0 [27.0–96.0] | 7–1133 |
|  | Radiotherapy | 7 | 79.1 ± 43.2 | 79.0 [46.0–110.0] | 17–151 |
| **Age** | Less than 50 | 24 | 57.6 ± 51.6 | 50.0 [17.3–81.3] | 3–183 |
|  | Greater than 50 | 43 | 123.8 ± 213.5 | 53.0 [28.0–104.0] | 10–1133 |
| **Distance** | Less than 200km | 42 | 102.9 ± 211.7 | 46.5 [22.0–83.3] | 7–1133 |
|  | Greter than 200km | 25 | 95.4 ± 92.4 | 53.0 [31.0–134.0] | 3–362 |
