# Supplementary material for: Prevalence and factors associated with treatment delay among colorectal cancer patients at Mulago National Referral Hospital and the Uganda Cancer Institute: A cross-sectional study
Source: PLoS One. 2026 Jul 17;21(7):e0353668. doi: 10.1371/journal.pone.0353668 (PMC13378963; doi:10.1371/journal.pone.0353668)
Supplement: Attachment — Submitted filename: Review commetns.docx [file pone.0353668.s003.docx]

Manuscript Number: PONE-D-25-56335 Title: Prevalence and Factors Associated with Treatment Delay Among Colorectal Cancer Patients at Mulago National Referral Hospital and the Uganda Cancer Institute: A Cross-Sectional Study

General Remarks

The manuscript discusses a major public health problem: the "triple threat" of rising colorectal cancer (CRC) rates, late diagnosis, and systemic problems in areas with few resources. The study offers significant insights into the Diagnosis-to-Treatment Interval (DTI) in Uganda, revealing a substantial “prevalence delay” of 70.1% and highlighting socioeconomic status (SES) as a key independent predictor. The research is clinically relevant and methodologically robust in its application of composite scores; however, numerous significant inconsistencies and statistical issues require revision prior to publication.

Major Changes

1. Ethical contradictions There is a direct contradiction regarding patient consent that needs to be made clear for ethical reasons:

• The authors say in the Methods section that "Written consent was obtained from all participants."

• But the Declarations section says that the study was "approved with waiver of consent."

• Recommendation: The authors need to make it clear which protocol they followed and make sure the whole manuscript is consistent.

2. Reference Groups and Statistical Stability. The sample size of N=67 causes some regression models to be unstable:

• The bivariate analysis of education levels employs "Postgraduate" as a reference group, comprising merely 2 participants (3.0% of the cohort). The authors themselves say that these results are "likely artefactual."

• Suggestion: The authors should combine educational categories, such as "College/University" and "Postgraduate," to make stronger reference groups for analysis.

3. Check the study's timeline The authors assert that data collection transpired from December 2024 to May 2025.

• Question: Since today is the current date, the authors should check to see if the May 2025 data is final or just a guess. If the study is still going on, the results must be clearly marked as preliminary.

Minor Changes

4. Table 1: Data Completeness

• Table 1 shows that 3 patients (4.5%) are missing data for "Clinical Stage."

• Suggestion: A short note should be added to the Results section or a footnote to Table 1 to explain why this data was not available (for example, because medical records were not complete).

5. Why SES Scoring is Important

• The post-hoc SES composite score incorporates "alternative medicine use" as a variable signifying susceptibility.

• Recommendation: Although this health behaviour is frequently associated with delays, the authors should succinctly elucidate in the Discussion why it was classified as a socioeconomic disadvantage instead of a cultural or clinical factor.

6. Improvement of Figure 1

• Figure 1 (a pie chart) shows "Delay" and "No Delay."

• Suggestion: To make it easier to read, the chart should have the exact percentages (70.1% and 29.9%) right on the visual parts to match the text.

7. Abbreviations • SES (Socioeconomic Status) and DTI (Diagnosis-to-Treatment Interval) should be added to the List of Abbreviations because they are important to the study's results.

Conclusion

This research offers essential evidence concerning the structural and financial impediments to oncological care in Uganda. This manuscript will be a strong candidate for publication if it fixes the ethical problems and improves the way the statistics are presented.
