# Supplementary material for: Prevalence and factors associated with treatment delay among colorectal cancer patients at Mulago National Referral Hospital and the Uganda Cancer Institute: A cross-sectional study
Source: PLoS One. 2026 Jul 17;21(7):e0353668. doi: 10.1371/journal.pone.0353668 (PMC13378963; doi:10.1371/journal.pone.0353668)
Supplement: Attachment — Submitted filename: Response to Reviewers_.docx [file pone.0353668.s005.docx]

**REBUTTAL LETTER**

We sincerely thank the Editor and the Reviewers for their careful evaluation of our manuscript and for the constructive comments and suggestions. We are grateful for the time and expertise invested in reviewing our work. The comments have been extremely helpful in improving the clarity, methodological transparency, and overall quality of the manuscript.

We have carefully revised the manuscript in response to all comments. Detailed responses to each point are provided below, and all corresponding changes have been made in the revised manuscript with tracked changes. Line numbers cited in this document refer to the revised manuscript.

**Reviewer #1: Preamble: Allow me to first thank the authors on this great work on an important topic of public health concern. Below are my review comments;-.
Introduction: The introduction does not clearly describe what is not known about the delay to initiate treatment for colo-rectal cancer (CRC) and the factors associated with this delay. The knowledge gap (scientific value) which the study intends to address should be clearly described. It is not enough to just mention that there are few studies. Even the few studies are about the causes not the factors associated.**

We thank the reviewer for this important comment. The Introduction has been revised to more clearly articulate the specific knowledge gap addressed by this study. We now explicitly state that, despite recognition that treatment delays are potentially modifiable contributors to poor colorectal cancer outcomes, there is limited context-specific evidence in Uganda quantifying diagnosis-to-treatment intervals and identifying factors associated with delayed treatment initiation. We further clarify that existing studies have largely focused on describing care pathways rather than systematically examining patient-level, clinicopathologic, and socioeconomic determinants of treatment delay for colorectal cancer.

Line 69-70

**Line 9 and 10: Epidemiologically, low screening results into less incidence as new cases are not detected. Can the authors explain how low screening contributes to the growing incidence of CRC?**

We thank the reviewer for this important epidemiological clarification. The reference to low screening coverage contributing to increasing colorectal cancer incidence has been removed from the Introduction to avoid conceptual inaccuracy.

Line 42-43

**Line 15 and 27: Use only abbreviation for CRC to ensure standard writing. This should be done throughout the whole manuscript.**

We thank the reviewer for this comment. The manuscript has been revised to ensure consistent use of the abbreviation CRC throughout. “Colorectal cancer” is now written in full at first mention, followed by the abbreviation, and CRC is used consistently thereafter across the entire manuscript.

**Methods: The methods section is not well written. There is a mix up of what should be included in the various sub-headings under the methods section.
There is need to consult the author guidelines while writing these sub-headings and ensure that all that is required according to the guidelines is addressed.
Specifically, the following should be addressed
i) Study design and setting: The study design is clearly mentioned but is not appropriate for the study question. A cross-sectional study does not appropriately answer the question of time to event (from diagnosis to initiation of treatment). Authors need to explain how they were able to measure this time using a cross-sectional study design.
The authors should justify why use two study sites (Mulago National Referral Hospital and Uganda Cancer Institute). The study setting should clearly describe relevant contextual information for an unfamiliar reader to understand. For example, how many patients (on average) were diagnosed with CRC at each of the two sites, what services are offered, who offers the services etc? Who funds the services? What is their catchment area? This will help to reader to appreciate the feasibility of the study as well as contextual understanding of the findings.**
We thank the reviewer for this comment. The Methods section has been revised to clarify how the diagnosis-to-treatment interval was measured in this retrospective cross-sectional study. All participants had already initiated first oncologic treatment at enrolment, allowing calculation of the interval between histological diagnosis and treatment initiation and estimation of the prevalence of treatment delay.

We have also expanded the description of the study setting to justify inclusion of both Mulago National Referral Hospital and the Uganda Cancer Institute. The revised manuscript now outlines the complementary roles, patient volumes, services offered, and national catchment of the two institutions, reflecting the standard public-sector colorectal cancer care pathway in Uganda and supporting the feasibility and contextual relevance of the study.

Lines 84-100

**The ethical considerations of the study should not be part of the section. They should be a separate section.**

This has been appropriately done. Thank you

Line 200-204

**ii) study population and sampling: This section should be chronologically and logically written. The study population is clearly described. Clearly describe how the sample size was determined and the any assumptions made. Explicitly outline the sampling procedure and the exclusion and inclusion criteria. Line 61 and 62, be clear on what prevalence was going to be estimated. Therefore, re-write and re-arrange this section for chronology and logical flow.
How did the sampling cater for contextual differences between MNRH and UCI? Proportionate sampling should have been done.**

We thank the reviewer for this detailed comment. The Study population and sampling subsection has been revised to improve chronological structure and methodological transparency. The revised manuscript now clearly describes eligibility and exclusion criteria, recruitment points along the colorectal cancer care pathway, and sample size determination for each study objective.

We have clarified that the prevalence estimated refers to the proportion of colorectal cancer patients experiencing treatment delay. Sample size estimation for this objective was based on the Kish–Leslie single population proportion formula with assumptions drawn from prior literature and application of a finite population correction informed by hospital records from both study sites.

For the objective of identifying factors associated with treatment delay, sample size was calculated using the Fleiss formula for comparison of proportions. The final sample size was taken as the larger of the estimates derived for the prevalence and factors objectives. The sampling procedure is now described as consecutive enrolment across Mulago National Referral Hospital and the Uganda Cancer Institute. Given the integrated referral pathway and frequent patient movement between institutions, strict proportionate sampling by site was not operationally feasible, and enrolment reflected routine patient flow across different stages of care.

Line 112-143

**iii) Data collection: The authors just mention that data was collected using patient interviews and medical record reviews. The authors should explicitly describe which kind of interviews, tools used with their validity and reliability. Which records were reviewed; outpatient forms or in-patient files, what information was extracted from the reviews and what tools were used if any?**

We thank the reviewer for this comment. The Data Collection section has been revised to explicitly describe the data sources, tools, and variables obtained through each method.

The revised manuscript now clarifies that both structured patient interviews and medical record review were required because no single source contained all information necessary for the study objectives. Clinical and treatment-related variables, including dates of histological diagnosis, treatment initiation, clinical stage, and type of first oncologic treatment, were obtained from patient records. In contrast, sociodemographic characteristics, health-seeking pathways, referral experiences, use of alternative medicine, and patient-reported barriers to treatment initiation were obtained through interviewer-administered patient interviews, as these data are not routinely documented in the medical chart files

Furthermore, most files had missing information and we had to use multiple sources to complete data collection.

We have also specified the types of records reviewed at each study site and described the use of structured questionnaires and standardized data abstraction forms, as well as cross-checking interview data against available records where applicable to improve completeness and consistency.

Line 147-162

**Line
iv) Data analysis: The definition of treatment delay as a primary outcome of analysis was derived from an Ethiopian study according to the citation 31. Isn’t there an internationally accepted definition of treatment delay for cancers? This needs to be explained further.**

Thank you for this comment. Although there is no universally agreed baseline for defining treatment delay, systematic reviews by Hannah et al. and Molenaar et al. (2021) show that most studies and clinical guidelines recommend treatment initiation within 2 to 7 weeks (median approximately 4.5 weeks) from pathological diagnosis. We used a recent study’s cut off (Wassie et al of 31 days, in an Ethiopian setting which is close to the Ugandan context. The median diagnosis-to-treatment interval in our cohort was 53 days, clearly exceeding these commonly cited benchmarks.

An explanation is needed why Poisson regression, a non-parametric test was used. 95% confidence interval should be revised to 95% level of confidence.

Thank you for this important methodological comment.

In this study, Poisson regression with robust variance estimation was used to analyse factors associated with treatment delay. This approach was selected because the outcome variable (treatment delay: yes/no) was binary and relatively common in the study population (prevalence 70.1%). Under these conditions, logistic regression can yield odds ratios that substantially overestimate prevalence ratios and are less straightforward to interpret in cross-sectional analyses.

Modified Poisson regression with robust standard errors allows direct estimation of prevalence ratios, which were the measures of association of interest in this study. This approach has been widely recommended for cross-sectional studies with common outcomes and is frequently used in epidemiological analyses for this reason. We have clarified this rationale in the Methods section.

With regard to confidence intervals, we have revised the manuscript to ensure consistent reporting of 95% confidence intervals (95% CI) throughout, corresponding to a 95% level of confidence.

References

1. Alternatives for logistic regression in cross-sectional studies: an empirical comparison of models that directly estimate the prevalence ratio <https://link.springer.com/article/10.1186/1471-2288-3-21>
2. A modified poisson regression approach to prospective studies with binary data Guangyong Zou PMID: 15033648 DOI: 10.1093/aje/kwh090

Line 165-178

Use of composite scores complicated the interpretation and understanding of the results. All the study set out to do was to measure time from CRC histological diagnosis and initiation of treatment. The appropriate method of analysis should have been survival analysis, a statistical measure of time to occurrence of the event or outcome of interest in this case initiation of treatment. The composite scores cannot ably measure the factors associated. Multiple regression should have catered for the multi-dimensional nature of the barriers to care.
Therefore, the statistics used in this study are not appropriate.

We thank the reviewer for this important comment. We agree that interpretation of composite scores can be challenging and have revised the manuscript to clarify their role in the analysis. Composite scores were used as exploratory summaries of related barriers to care to reduce dimensionality and facilitate interpretation, but were not intended to replace analysis of individual factors associated with treatment delay. We have clarified this in the Methods section and de-emphasised composite score findings in the Results.

With regard to the suggestion of survival analysis, we note that the primary objectives of the study were to estimate the prevalence of treatment delay and to identify factors associated with delay as a binary outcome. The diagnosis-to-treatment interval was summarised descriptively, while inferential analyses focused on whether delay occurred rather than modelling the time to treatment initiation. Given these objectives and the cross-sectional design, we considered prevalence-based regression approaches to be appropriate for the primary analyses.

We have revised the manuscript to better align the presentation of the analyses with the study objectives and to clarify the analytical choices made.

Line 180-198, Line 194 -197

**Results
The results are well presented in both narratives and tables. However, the following issues are noted;
1. The narratives are repetitions of what is already presented in the tables.
2. It is not clear which results are from patient interviews and from review of records.
Tables 3 and 4: The results from analysis of composite scores should be presented in narratives instead of table.**

We thank the reviewer for this comment. We have revised the Results section to reduce repetition between the narrative text and the tables, ensuring that the text now focuses on interpretation and key patterns rather than restating numerical values already presented in the tables.

In response to the concern regarding composite scores, we have clarified their purpose and revised their presentation. Composite scores were used as exploratory summaries to capture the multidimensional nature of barriers to care and were not intended to replace analyses of individual factors. In line with the reviewer’s suggestion, detailed composite score results are now presented in the Supplementary Material, with only a concise narrative summary retained in the main Results section.

We have also clarified that results are derived from combined information obtained through patient interviews and medical record review, with interviews complementing clinical records where documentation was incomplete. We therefore did not seek for specific data from files or interviews, but rather used both sources complementarily, because of missing data from most of the file charts.

Lines 206-212, 259-266, 277-284

Discussion
The discussion section should start by discussing the key findings from the study, mention their contribution to the current available body of knowledge before comparing them with the available literature. The discussion has significant repeatition of results (results should not be repeated in the discussion section but should be discussed).
The discussion lacks clear strengths and weaknesses/limitations of the study as well as implications of the study results and their possible explanations. For example, why a low sample size yet two study sites were used? Consecutive sampling, a non-probability sampling technique should be acknowledged given the potential bias within such sample..

Thank you so much for this comment. These corrections have been appropriately made in the discussion and limitations paragraph

Line 288-301, 367-378

**Reviewer #2:** Major Changes
1. Ethical contradictions There is a direct contradiction regarding patient consent that needs to be made clear for ethical reasons:
• The authors say in the Methods section that "Written consent was obtained from all participants."
• But the Declarations section says that the study was "approved with waiver of consent."
• Recommendation: The authors need to make it clear which protocol they followed and make sure the whole manuscript is consistent.

We thank the reviewer for identifying this inconsistency. We acknowledge that the manuscript contained contradictory statements regarding informed consent.

We have revised the manuscript to ensure consistency throughout. The study was approved by the relevant ethics committee, and written informed consent was obtained from all participants prior to enrolment. References to a waiver of consent have been removed from the Declarations section.

The Methods and Declarations sections now accurately reflect the ethical procedures followed in the study.

Line 200-204

**2. Reference Groups and Statistical Stability. The sample size of N=67 causes some regression models to be unstable:
• The bivariate analysis of education levels employs "Postgraduate" as a reference group, comprising merely 2 participants (3.0% of the cohort). The authors themselves say that these results are "likely artefactual."
• Suggestion: The authors should combine educational categories, such as "College/University" and "Postgraduate," to make stronger reference groups for analysis.**

,We thank the reviewer for highlighting the concern regarding statistical stability in the education-level analyses. We agree that using “postgraduate” as the reference category in the original bivariate model was inappropriate because the postgraduate group included only two participants, which could yield unstable estimates and potentially misleading inference.

Revisions made (education reclassification): In response, we reclassified education into three analytically stable and conceptually meaningful categories: (i) primary or less (no formal education + primary), (ii) secondary, and (iii) tertiary (college/university + postgraduate). Tertiary education was used as the reference category. This change was made specifically to address small cell sizes and improve the stability and interpretability of regression estimates. The revised categorisation is reflected in the updated Results table and described in the Methods.

Re-analysis performed (modified Poisson regression): We re-ran the education analysis using modified Poisson regression with robust variance estimation (Poisson distribution with log link and sandwich/robust standard errors), consistent with our approach for estimating prevalence ratios in this cross-sectional study with a common outcome.

Updated results: After reclassification and re-analysis, education level was not significantly associated with treatment delay in bivariate analysis (overall test p = 0.465). Compared with tertiary education, the prevalence ratio for treatment delay was PR = 0.98 (95% CI: 0.71–1.36; p = 0.913) for primary or less education, and PR = 0.64 (95% CI: 0.37–1.09; p = 0.099) for secondary education. These findings indicate no statistically significant differences in treatment delay prevalence across the reclassified education categories.

Lines 242-246

Manuscript updates to ensure consistency and appropriate interpretation:

1. Table updates: The education rows in the bivariate analysis table were revised to reflect the collapsed categories and the updated estimates. The previous “postgraduate” reference group was removed. A table footnote now explicitly states the reclassification (primary or less; secondary; tertiary) undertaken for statistical stability.
2. Results text: We removed the prior interpretation of education as “protective” and removed language describing results as “artefactual.” The Results section now reports the updated bivariate findings with appropriate caution and without over-interpretation.
3. Methods (Data analysis): We added a clarifying sentence noting that education categories were collapsed prior to regression analyses to improve statistical stability due to small cell sizes.
4. Discussion: We revised the Discussion to align with the updated results, stating that education alone was not independently associated with treatment delay after reclassification, and emphasising that single socioeconomic indicators may be limited when interpreted in isolation.
5. Limitations: We added a brief cautionary statement noting that education is an imperfect proxy for socioeconomic position and that findings based on individual socioeconomic indicators should be interpreted cautiously, while retaining focus on the multidimensional socioeconomic vulnerability composite, which remained associated with delay in multivariable analysis.

Line 254-256

Line 369-371

**3. Check the study's timeline The authors assert that data collection transpired from December 2024 to May 2025.
• Question: Since today is the current date, the authors should check to see if the May 2025 data is final or just a guess. If the study is still going on, the results must be clearly marked as preliminary.**
We thank the reviewer for this request for clarification. We would like to clarify that this study was a hospital-based cross-sectional study conducted between December 2024 and May 2025. During this period, patients with colorectal cancer who had already initiated first oncologic treatment were identified, and relevant clinical dates were retrospectively extracted from medical records.

The dates December 2024 to May 2025 therefore represent the fixed data collection period, not a projected or ongoing timeline. No follow-up or prospective observation was undertaken, and all analyses were based on data available at the time of enrolment. The results presented are based on complete, final data, not preliminary findings. We have revised the Methods section to clarify this point.

Lines 84-90.

**Minor Changes
4. Table 1: Data Completeness
• Table 1 shows that 3 patients (4.5%) are missing data for "Clinical Stage."
• Suggestion: A short note should be added to the Results section or a footnote to Table 1 to explain why this data was not available (for example, because medical records were not complete).**

We thank the reviewer for this observation. We have clarified this in the manuscript by adding a note indicating that clinical stage data were missing for three patients due to incomplete documentation in the medical records at the time of data abstraction.

Line 225-226

**5. Why SES Scoring is Important
• The post-hoc SES composite score incorporates "alternative medicine use" as a variable signifying susceptibility.
• Recommendation: Although this health behaviour is frequently associated with delays, the authors should succinctly elucidate in the Discussion why it was classified as a socioeconomic disadvantage instead of a cultural or clinical factor.**

We thank the reviewer for this thoughtful comment and agree that clarification of the conceptual rationale for including alternative medicine use in the socioeconomic vulnerability composite is important.

We have revised the Discussion to explicitly explain why use of alternative or traditional medicine was conceptualised as a marker of socioeconomic vulnerability rather than a purely cultural or clinical factor. In the Ugandan context and similar low-resource settings, reliance on alternative medicine is frequently shaped by structural constraints such as financial barriers, geographic inaccessibility of biomedical services, and delays within the formal health system, rather than cultural preference alone.

We have added a concise explanation in the Discussion, supported by relevant references, to clarify that inclusion of alternative medicine use in the SES composite reflects constrained access to timely and affordable biomedical care, which is directly relevant to treatment delay. These revisions are intended to avoid cultural misinterpretation and to strengthen the conceptual framework underlying the SES composite.

Line 351-357

**6. Improvement of Figure 1
• Figure 1 (a pie chart) shows "Delay" and "No Delay."
• Suggestion: To make it easier to read, the chart should have the exact percentages (70.1% and 29.9%) right on the visual parts to match the text.**

We thank the reviewer for this helpful suggestion. We have revised Figure 1 to display the exact percentages for both categories (treatment delay and no delay) directly on the pie chart.

**7. Abbreviations • SES (Socioeconomic Status) and DTI (Diagnosis-to-Treatment Interval) should be added to the List of Abbreviations because they are important to the study's results.**

We thank the reviewer for this suggestion. We have revised the manuscript to include SES (Socioeconomic Status) and DTI (Diagnosis-to-Treatment Interval) in the List of Abbreviations, ensuring consistency and clarity throughout the manuscript.

Thank you!

With gratitude

Brian Kasagga

On behalf of all authors
