# Supplementary material for: Prevalence and factors associated with treatment delay among colorectal cancer patients at Mulago National Referral Hospital and the Uganda Cancer Institute: A cross-sectional study
Source: PLoS One. 2026 Jul 17;21(7):e0353668. doi: 10.1371/journal.pone.0353668 (PMC13378963; doi:10.1371/journal.pone.0353668)
Supplement: Peer review history

# Peer review: Decision Letter 0

**DOI:** 10.1371/journal.pone.0353668.r001

**Authors:** Aloysius Gonzaga Mubuuke

21 Jan 2026

PONE-D-25-56335

Prevalence and Factors Associated with Treatment Delay Among Colorectal Cancer Patients at Mulago National Referral Hospital and the Uganda Cancer Institute: A Cross-Sectional Study

PLOS One

Dear Dr. Kasagga,

Thank you for submitting your manuscript to PLOS ONE. After careful consideration, we feel that it has merit but does not fully meet PLOS ONE’s publication criteria as it currently stands. Therefore, we invite you to submit a revised version of the manuscript that addresses the points raised during the review process.

Please submit your revised manuscript by Mar 07 2026 11:59PM. If you will need more time than this to complete your revisions, please reply to this message or contact the journal office at plosone@plos.org. When you're ready to submit your revision, log on to https://www.editorialmanager.com/pone/ and select the 'Submissions Needing Revision' folder to locate your manuscript file.

Please include the following items when submitting your revised manuscript:

- A letter that responds to each point raised by the academic editor and reviewer(s). You should upload this letter as a separate file labeled 'Response to Reviewers'.
- A marked-up copy of your manuscript that highlights changes made to the original version. You should upload this as a separate file labeled 'Revised Manuscript with Track Changes'.
- An unmarked version of your revised paper without tracked changes. You should upload this as a separate file labeled 'Manuscript'.

If you would like to make changes to your financial disclosure, please include your updated statement in your cover letter. Guidelines for resubmitting your figure files are available below the reviewer comments at the end of this letter.

If applicable, we recommend that you deposit your laboratory protocols in protocols.io to enhance the reproducibility of your results. Protocols.io assigns your protocol its own identifier (DOI) so that it can be cited independently in the future. For instructions see: https://journals.plos.org/plosone/s/submission-guidelines#loc-laboratory-protocols. Additionally, PLOS ONE offers an option for publishing peer-reviewed Lab Protocol articles, which describe protocols hosted on protocols.io. Read more information on sharing protocols at https://plos.org/protocols?utm_medium=editorial-email&utm_source=authorletters&utm_campaign=protocols.

We look forward to receiving your revised manuscript.

Kind regards,

Aloysius Gonzaga Mubuuke

Academic Editor

PLOS One

**Journal Requirements:**

1. When submitting your revision, we need you to address these additional requirements.

Please ensure that your manuscript meets PLOS ONE's style requirements, including those for file naming. The PLOS ONE style templates can be found at

https://journals.plos.org/plosone/s/file?id=wjVg/PLOSOne_formatting_sample_main_body.pdf and

https://journals.plos.org/plosone/s/file?id=ba62/PLOSOne_formatting_sample_title_authors_affiliations.pdf

2. Please note that your Data Availability Statement is currently missing the DOI/accession number of each dataset and a direct link to access each database. If your manuscript is accepted for publication, you will be asked to provide these details on a very short timeline. We therefore suggest that you provide this information now, though we will not hold up the peer review process if you are unable.

3. Your ethics statement should only appear in the Methods section of your manuscript. If your ethics statement is written in any section besides the Methods, please delete it from any other section.

4. Please upload a new copy of Figure as the detail is not clear. Please follow the link for more information:  https://journals.plos.org/plosone/s/figures

5. Please include captions for your Supporting Information files at the end of your manuscript, and update any in-text citations to match accordingly. Please see our Supporting Information guidelines for more information: http://journals.plos.org/plosone/s/supporting-information.

6. If the reviewer comments include a recommendation to cite specific previously published works, please review and evaluate these publications to determine whether they are relevant and should be cited. There is no requirement to cite these works unless the editor has indicated otherwise.

[Note: HTML markup is below. Please do not edit.]

Reviewers' comments:

Reviewer's Responses to Questions

**Comments to the Author**

1. Is the manuscript technically sound, and do the data support the conclusions?

The manuscript must describe a technically sound piece of scientific research with data that supports the conclusions. Experiments must have been conducted rigorously, with appropriate controls, replication, and sample sizes. The conclusions must be drawn appropriately based on the data presented.

Reviewer #1: Partly

Reviewer #2: Yes

**********

2. Has the statistical analysis been performed appropriately and rigorously?

Reviewer #1: No

Reviewer #2: I Don't Know

**********

3. Have the authors made all data underlying the findings in their manuscript fully available?

The PLOS Data policy requires authors to make all data underlying the findings described in their manuscript fully available without restriction, with rare exception (please refer to the Data Availability Statement in the manuscript PDF file). The data should be provided as part of the manuscript or its supporting information, or deposited to a public repository. For example, in addition to summary statistics, the data points behind means, medians and variance measures should be available. If there are restrictions on publicly sharing data—e.g. participant privacy or use of data from a third party—those must be specified.

Reviewer #1: No

Reviewer #2: Yes

**********

4. Is the manuscript presented in an intelligible fashion and written in standard English?

PLOS ONE does not copyedit accepted manuscripts, so the language in submitted articles must be clear, correct, and unambiguous. Any typographical or grammatical errors should be corrected at revision, so please note any specific errors here.

Reviewer #1: Yes

Reviewer #2: Yes

**********

5. Review Comments to the Author

Please use the space provided to explain your answers to the questions above. You may also include additional comments for the author, including concerns about dual publication, research ethics, or publication ethics. (Please upload your review as an attachment if it exceeds 20,000 characters)

**Reviewer #1:**  Preamble: Allow me to first thank the authors on this great work on an important topic of public health concern. Below are my review comments;

Introduction: The introduction does not clearly describe what is not known about the delay to initiate treatment for colo-rectal cancer (CRC) and the factors associated with this delay. The knowledge gap (scientific value) which the study intends to address should be clearly described. It is not enough to just mention that there are few studies. Even the few studies are about the causes not the factors associated.

Line 9 and 10: Epidemiologically, low screening results into less incidence as new cases are not detected. Can the authors explain how low screening contributes to the growing incidence of CRC?

Line 15 and 27: Use only abbreviation for CRC to ensure standard writing. This should be done throughout the whole manuscript.

Methods: The methods section is not well written. There is a mix up of what should be included in the various sub-headings under the methods section.

There is need to consult the author guidelines while writing these sub-headings and ensure that all that is required according to the guidelines is addressed.

Specifically, the following should be addressed

i) Study design and setting: The study design is clearly mentioned but is not appropriate for the study question. A cross-sectional study does not appropriately answer the question of time to event (from diagnosis to initiation of treatment). Authors need to explain how they were able to measure this time using a cross-sectional study design.

The authors should justify why use two study sites (Mulago National Referral Hospital and Uganda Cancer Institute). The study setting should clearly describe relevant contextual information for an unfamiliar reader to understand. For example, how many patients (on average) were diagnosed with CRC at each of the two sites, what services are offered, who offers the services etc? Who funds the services? What is their catchment area? This will help to reader to appreciate the feasibility of the study as well as contextual understanding of the findings.

The ethical considerations of the study should not be part of the section. They should be a separate section.

ii) study population and sampling: This section should be chronologically and logically written. The study population is clearly described. Clearly describe how the sample size was determined and the any assumptions made. Explicitly outline the sampling procedure and the exclusion and inclusion criteria. Line 61 and 62, be clear on what prevalence was going to be estimated. Therefore, re-write and re-arrange this section for chronology and logical flow.

How did the sampling cater for contextual differences between MNRH and UCI? Proportionate sampling should have been done.

iii) Data collection: The authors just mention that data was collected using patient interviews and medical record reviews. The authors should explicitly describe which kind of interviews, tools used with their validity and reliability. Which records were reviewed; outpatient forms or in-patient files, what information was extracted from the reviews and what tools were used if any?

iv) Data analysis: The definition of treatment delay as a primary outcome of analysis was derived from an Ethiopian study according to the citation 31. Isn’t there an internationally accepted definition of treatment delay for cancers? This needs to be explained further.

An explanation is needed why Poisson regression , a non-parametric test was used. 95% confidence interval should be revised to 95% level of confidence.

Use of composite scores complicated the interpretation and understanding of the results. All the study set out to do was to measure time from CRC histological diagnosis and initiation of treatment. The appropriate method of analysis should have been survival analysis, a statistical measure of time to occurrence of the event or outcome of interest in this case initiation of treatment.

The composite scores cannot ably measure the factors associated. Multiple regression should have catered for the multi-dimensional nature of the barriers to care.

Therefore, the statistics used in this study are not appropriate.

Results

The results are well presented in both narratives and tables. However, the following issues are noted;

1. The narratives are repetitions of what is already presented in the tables.

2. It is not clear which results are from patient interviews and from review of records.

Tables 3 and 4: The results from analysis of composite scores should be presented in narratives instead of table.

Discussion

The discussion section should start by discussing the key findings from the study, mention their contribution to the current available body of knowledge before comparing them with the available literature. The discussion has significant repeatition of results (results should not be repeated in the discussion section but should be discussed).

The discussion lacks clear strengths and weaknesses/limitations of the study as well as implications of the study results and their possible explanations. For example, why a low sample size yet two study sites were used? Consecutive sampling, a non-probability sampling technique should be acknowledged given the potential bias within such sample.

**Reviewer #2:** Major Changes

1. Ethical contradictions There is a direct contradiction regarding patient consent that needs to be made clear for ethical reasons:

• The authors say in the Methods section that "Written consent was obtained from all participants."

• But the Declarations section says that the study was "approved with waiver of consent."

• Recommendation: The authors need to make it clear which protocol they followed and make sure the whole manuscript is consistent.

2. Reference Groups and Statistical Stability. The sample size of N=67 causes some regression models to be unstable:

• The bivariate analysis of education levels employs "Postgraduate" as a reference group, comprising merely 2 participants (3.0% of the cohort). The authors themselves say that these results are "likely artefactual."

• Suggestion: The authors should combine educational categories, such as "College/University" and "Postgraduate," to make stronger reference groups for analysis.

3. Check the study's timeline The authors assert that data collection transpired from December 2024 to May 2025.

• Question: Since today is the current date, the authors should check to see if the May 2025 data is final or just a guess. If the study is still going on, the results must be clearly marked as preliminary.

Minor Changes

4. Table 1: Data Completeness

• Table 1 shows that 3 patients (4.5%) are missing data for "Clinical Stage."

• Suggestion: A short note should be added to the Results section or a footnote to Table 1 to explain why this data was not available (for example, because medical records were not complete).

5. Why SES Scoring is Important

• The post-hoc SES composite score incorporates "alternative medicine use" as a variable signifying susceptibility.

• Recommendation: Although this health behaviour is frequently associated with delays, the authors should succinctly elucidate in the Discussion why it was classified as a socioeconomic disadvantage instead of a cultural or clinical factor.

6. Improvement of Figure 1

• Figure 1 (a pie chart) shows "Delay" and "No Delay."

• Suggestion: To make it easier to read, the chart should have the exact percentages (70.1% and 29.9%) right on the visual parts to match the text.

7. Abbreviations • SES (Socioeconomic Status) and DTI (Diagnosis-to-Treatment Interval) should be added to the List of Abbreviations because they are important to the study's results.

Conclusion

This research offers essential evidence concerning the structural and financial impediments to oncological care in Uganda. This manuscript will be a strong candidate for publication if it fixes the ethical problems and improves the way the statistics are presented.

**********

6. PLOS authors have the option to publish the peer review history of their article (what does this mean?). If published, this will include your full peer review and any attached files.

If you choose “no”, your identity will remain anonymous but your review may still be made public.

**Do you want your identity to be public for this peer review?**  For information about this choice, including consent withdrawal, please see our Privacy Policy.

Reviewer #1: No

Reviewer #2: No

**********

[NOTE: If reviewer comments were submitted as an attachment file, they will be attached to this email and accessible via the submission site. Please log into your account, locate the manuscript record, and check for the action link "View Attachments". If this link does not appear, there are no attachment files.]

To ensure your figures meet our technical requirements, please review our figure guidelines: https://journals.plos.org/plosone/s/figures

You may also use PLOS’s free figure tool, NAAS, to help you prepare publication quality figures: https://journals.plos.org/plosone/s/figures#loc-tools-for-figure-preparation.

NAAS will assess whether your figures meet our technical requirements by comparing each figure against our figure specifications.

Attachment Submitted filename: Review commetns.docx

# Author comment: Author response to Decision Letter 1

**DOI:** 10.1371/journal.pone.0353668.r002

12 Mar 2026

The response to the reviewers letter has been appropriately attached.

Thank you, please.

Attachment Submitted filename: Response to Reviewers_.docx

# Peer review: Decision Letter 1

**DOI:** 10.1371/journal.pone.0353668.r003

**Authors:** Aloysius Gonzaga Mubuuke, Aloysius Gonzaga Mubuuke

29 Jun 2026

Prevalence and Factors Associated with Treatment Delay Among Colorectal Cancer Patients at Mulago National Referral Hospital and the Uganda Cancer Institute: A Cross-Sectional Study

PONE-D-25-56335R1

Dear Dr. Kasagga,

We’re pleased to inform you that your manuscript has been judged scientifically suitable for publication and will be formally accepted for publication once it meets all outstanding technical requirements.

Within one week, you’ll receive an e-mail detailing the required amendments. When these have been addressed, you’ll receive a formal acceptance letter and your manuscript will be scheduled for publication.

An invoice will be generated when your article is formally accepted. Please note, if your institution has a publishing partnership with PLOS and your article meets the relevant criteria, all or part of your publication costs will be covered. Please make sure your user information is up-to-date by logging into Editorial Manager at Editorial Manager® and clicking the ‘Update My Information' link at the top of the page. For questions related to billing, please contact billing support.

If your institution or institutions have a press office, please notify them about your upcoming paper to help maximize its impact. If they’ll be preparing press materials, please inform our press team as soon as possible -- no later than 48 hours after receiving the formal acceptance. Your manuscript will remain under strict press embargo until 2 pm Eastern Time on the date of publication. For more information, please contact onepress@plos.org.

Kind regards,

Aloysius Gonzaga Mubuuke

Academic Editor

PLOS One

Additional Editor Comments (optional):

All revisions have been addressed

Reviewers' comments:

Reviewer's Responses to Questions

**Comments to the Author**

1. If the authors have adequately addressed your comments raised in a previous round of review and you feel that this manuscript is now acceptable for publication, you may indicate that here to bypass the “Comments to the Author” section, enter your conflict of interest statement in the “Confidential to Editor” section, and submit your "Accept" recommendation.

Reviewer #1: All comments have been addressed

Reviewer #2: All comments have been addressed

**********

2. Is the manuscript technically sound, and do the data support the conclusions?

The manuscript must describe a technically sound piece of scientific research with data that supports the conclusions. Experiments must have been conducted rigorously, with appropriate controls, replication, and sample sizes. The conclusions must be drawn appropriately based on the data presented.

Reviewer #1: Yes

Reviewer #2: Yes

**********

3. Has the statistical analysis been performed appropriately and rigorously?

Reviewer #1: Yes

Reviewer #2: I Don't Know

**********

4. Have the authors made all data underlying the findings in their manuscript fully available?

The PLOS Data policy requires authors to make all data underlying the findings described in their manuscript fully available without restriction, with rare exception (please refer to the Data Availability Statement in the manuscript PDF file). The data should be provided as part of the manuscript or its supporting information, or deposited to a public repository. For example, in addition to summary statistics, the data points behind means, medians and variance measures should be available. If there are restrictions on publicly sharing data—e.g. participant privacy or use of data from a third party—those must be specified.

Reviewer #1: Yes

Reviewer #2: Yes

**********

5. Is the manuscript presented in an intelligible fashion and written in standard English?

PLOS ONE does not copyedit accepted manuscripts, so the language in submitted articles must be clear, correct, and unambiguous. Any typographical or grammatical errors should be corrected at revision, so please note any specific errors here.

Reviewer #1: Yes

Reviewer #2: Yes

**********

6. Review Comments to the Author

Please use the space provided to explain your answers to the questions above. You may also include additional comments for the author, including concerns about dual publication, research ethics, or publication ethics. (Please upload your review as an attachment if it exceeds 20,000 characters)

Reviewer #1: Dear Author(s),

Thank you for taking time to revise the manuscript according to the comments raised during the review. I am happy that all the issues/concerns raised during the review were tkaen seriously and addressed.

Reviewer #2: The manuscript discusses a major public health problem: the "triple threat" of rising colorectal cancer (CRC) rates, late diagnosis, and systemic problems in areas with few resources. The study offers significant insights into the Diagnosis-to-Treatment Interval (DTI) in Uganda, revealing a substantial “prevalence delay” of 70.1% and highlighting socioeconomic status (SES) as a key independent predictor. The research is clinically relevant and methodologically robust in its application of composite scores; however, numerous significant inconsistencies and statistical issues require revision prior to publication.

The authors addressed all my comments. I have no further comments now. Thank you

**********

7. PLOS authors have the option to publish the peer review history of their article (what does this mean?). If published, this will include your full peer review and any attached files.

If you choose “no”, your identity will remain anonymous but your review may still be made public.

**Do you want your identity to be public for this peer review?**  For information about this choice, including consent withdrawal, please see our Privacy Policy.

Reviewer #1: No

Reviewer #2: No

**********

# Editor report: Acceptance letter

**DOI:** 10.1371/journal.pone.0353668.r004

**Authors:** Aloysius Gonzaga Mubuuke, Aloysius Gonzaga Mubuuke

PONE-D-25-56335R1

PLOS One

Dear Dr. Kasagga,

I'm pleased to inform you that your manuscript has been deemed suitable for publication in PLOS One. Congratulations! Your manuscript is now being handed over to our production team.

At this stage, our production department will prepare your paper for publication. This includes ensuring the following:

* All references, tables, and figures are properly cited

* All relevant supporting information is included in the manuscript submission,

* There are no issues that prevent the paper from being properly typeset

You will receive further instructions from the production team, including instructions on how to review your proof when it is ready. Please keep in mind that we are working through a large volume of accepted articles, so please give us a few days to review your paper and let you know the next and final steps.

Lastly, if your institution or institutions have a press office, please let them know about your upcoming paper now to help maximize its impact. If they'll be preparing press materials, please inform our press team within the next 48 hours. Your manuscript will remain under strict press embargo until 2 pm Eastern Time on the date of publication. For more information, please contact onepress@plos.org.

You will receive an invoice from PLOS for your publication fee after your manuscript has reached the completed accept phase. If you receive an email requesting payment before acceptance or for any other service, this may be a phishing scheme. Learn how to identify phishing emails and protect your accounts at https://explore.plos.org/phishing.

If we can help with anything else, please email us at customercare@plos.org.

Thank you for submitting your work to PLOS ONE and supporting open access.

Kind regards,

PLOS ONE Editorial Office Staff

on behalf of

Dr. Aloysius Gonzaga Mubuuke

Academic Editor

PLOS One
